# Effectiveness and safety of edoxaban *versus* warfarin in patients with nonvalvular atrial fibrillation: a systematic review and meta-analysis of observational studies

**DOI:** 10.3389/fphar.2023.1276491

**Published:** 2023-11-16

**Authors:** Mohammed M. Alsultan, Abdullah K. Alahmari, Mansour A. Mahmoud, Ziyad S. Almalki, Wafa Alzlaiq, Faisal Alqarni, Fahad Alsultan, Nehad Jaser Ahmed, Ahmed O. Alenazi, Lucas Scharf, Jeff Jianfei Guo

**Affiliations:** ^1^ Department of Pharmacy Practice, College of Clinical Pharmacy, Imam Abdulrahman Bin Faisal University, Dammam, Saudi Arabia; ^2^ Department of Clinical Pharmacy, College of Pharmacy, Prince Sattam Bin Abdulaziz University, Al-Kharj, Saudi Arabia; ^3^ Department of Pharmacy Practice, College of Pharmacy, Taibah University, Al-Madinah Al-Munawara, Saudi Arabia; ^4^ Department of Pharmacy, Security Forces Hospital, Riyadh, Saudi Arabia; ^5^ College of Medicine, King Saud University, Riyadh, Saudi Arabia; ^6^ Pharmaceutical Care Department, Ministry of the National Guard-Health Affairs, Dammam, Saudi Arabia; ^7^ King Abdullah International Medical Research Center, Riyadh, Saudi Arabia; ^8^ King Saud Bin Abdulaziz University for Health Sciences, Riyadh, Saudi Arabia; ^9^ James L Winkle College of Pharmacy, University of Cincinnati, Cincinnati, OH, United States

**Keywords:** atrial fibrillation, warfarin, DOACs, edoxaban, safety and effectiveness

## Abstract

**Background:** Atrial fibrillation (AF) is the most prevalent cardiac arrhythmia type. Patients with AF are often administered anticoagulants to reduce the risk of ischemic stroke due to an irregular heartbeat. We evaluated the efficacy and safety of edoxaban *versus* warfarin in patients with nonvalvular AF by conducting an updated meta-analysis of real-world studies.

**Methods:** In this comprehensive meta-analysis, we searched two databases, PubMed and EMBASE, and included retrospective cohort observational studies that compared edoxaban with warfarin in patients with nonvalvular AF from 1 January 2009, to 30 September 2023. The effectiveness and safety outcomes were ischemic stroke and major bleeding, respectively. In the final analysis, six retrospective observational studies involving 87,236 patients treated with warfarin and 40,933 patients treated with edoxaban were included. To analyze the data, we used a random-effects model to calculate the hazard ratio (HR).

**Results:** Patients treated with edoxaban had a significantly lower risk of ischemic stroke [hazard ratio (HR) = 0.66; 95% confidence interval (CI) = 0.61–0.70; *p* < 0.0001] and major bleeding (HR = 0.58; 95% CI = 0.49–0.69; *p* < 0.0001) than those treated with warfarin. The sensitivity analysis results for ischemic stroke and major bleeding were as follows: HR = 0.66; 95% CI = 0.61–0.70; *p* < 0.0001 and HR = 0.58; 95% CI = 0.49–0.69; *p* < 0.0001, respectively.

**Conclusion:** Our findings revealed that edoxaban performed better than warfarin against major bleeding and ischemic stroke.

## 1 Introduction

The prevalence of atrial fibrillation (AF) is expected to rise globally in the coming years, with over 12 million cases expected among the population in the United States (US) and Europe by 2030 (Colilla et al., 2013; Krijthe et al., 2013; Lippi et al., 2021). In the Asian Region, the prevalence of AF was 0.79% in 2020 and projected to reach 5.4% by 2050 (Inoue et al., 2009; Chao et al., 2018; Kim et al., 2018). The irregular movement of blood from the atria to the ventricles can lead to blood clots and increase the risk of stroke (Goldstein et al., 2011). Furthermore, there may be a related risk between AF disease and cognitive function damage (Gallinoro et al., 2019). Patients with AF may benefit from therapy with the left atrial appendage occlusion (LAAO) approach to manage the increased bleeding risk (Cimmino et al., 2021). There are various forms of AF, including paroxysmal, chronic, long-lasting, permanent, and nonvalvular. AF without rheumatic mitral stenosis, biological or mechanical heart valve replacement, or mitral valve repair is known as nonvalvular AF (NVAF) (January et al., 2014; Heidbuche et al., 2015). Warfarin is a Vitamin K antagonist (VKA) that can stop strokes from happening, and it has long been the treatment of choice (Kuruvilla and Gurk-Turner, 2001; Zirlik and Bode, 2017). However, over the past 10 years, the US Food and Drug Administration (FDA) has approved four direct oral anticoagulants (DOACs) for NVAF: apixaban, dabigatran, rivaroxaban, and edoxaban. All DOACs were less likely than warfarin to interact with other medications and foods. Therefore, they do not require international normalized ratio (INR) monitoring after their use (Harter et al., 2015). In addition, the American Heart Association/American College of Cardiology (AHA/ACC) and the European Society of Cardiology (ESC) both suggest DOACs as the first line of treatment for ischemic stroke prevention in those diagnosed with NVAF (Heidbuche et al., 2015; J et al., 2019).

The ENGAGE AF-TIMI 48 trial showed that both once-daily regimens of edoxaban (60 and 30 mg) were non-inferior to warfarin with respect to the prevention of stroke or systemic embolism and were associated with significantly lower rates of bleeding and death from cardiovascular causes (Giugliano et al., 2013). In a systematic review and meta-analysis, Bai et al. found that rivaroxaban was comparable to warfarin for significant bleeding, and superior to dabigatran in preventing stroke and thromboembolism in patients with AF (Bai et al., 2017a).

Another meta-analysis of real-world studies found that the risk of thromboembolism or stroke associated with apixaban was similar to that associated with dabigatran, rivaroxaban, and edoxaban. The authors also found that apixaban was safer than warfarin in terms of the risk of major bleeding (Bai et al., 2017b). Dabigatran is comparable to warfarin in preventing ischemic stroke in patients with NVAF. However, dabigatran is associated with a lower incidence of cerebral bleeding than warfarin (Romanelli et al., 2016). Only a few meta-analyses have compared the safety and efficacy of edoxaban to warfarin, and they used network meta-analysis designs (Kim et al., 2021), meta-analyses of randomized controlled trials (Liang et al., 2021), and one meta-analysis that concentrated on individuals with different creatinine clearance (CrCl) values (Wang et al., 2023). However, recently, new cohort observational studies have not been included in previous meta-analyses. Observational data not only provide vital additional information to randomized controlled trials but also represent the most reliable data or perhaps the sole source of available evidence for some clinical concerns. Additionally, because observational studies are conducted in a more realistic environment than randomized controlled trials, which typically involve restricted populations receiving highly standardized care, their findings may be more directly applicable to the general population (Metelli and Chaimani, 2020). Therefore, we aimed to assess the effectiveness and safety of edoxaban and warfarin in patients with NVAF through an updated meta-analysis of real-world studies.

## 2 Methods

### 2.1 Search strategy

In this study, the systematic review was utilized in accordance with the Preferred Reporting Items for Systematic Review and Meta- Analysis (PRISMA) guidelines (Moher et al., 2009). We performed a comprehensive search for observational studies that compared edoxaban with warfarin between 1 January 2009, and 30 September 2023. Two databases were searched: PubMed and EMBASE. The title and abstract in both databases were searched using the following terms: “atrial fibrillation,” “AF,” “Warfarin,” and “Edoxaban.” This analysis included observational retrospective cohort studies on human subjects written in English.

### 2.2 Inclusion criteria

The study included only retrospective observational cohort studies that compared edoxaban and warfarin in patients diagnosed with NVAF. Randomized clinical trials, reviews, abstracts, editorials, case reports, case series, prospective studies, non-human studies, and cost analyses were excluded. Study outcomes included effectiveness (ischemic stroke) and safety (major bleeding).

### 2.3 Data extraction and quality assessment

Two authors independently extracted the articles and any differences between them were addressed by a third author. Data were extracted from included studies. The collected data included the names of the authors of the studies, the year of publication, the locations of these studies, and the sample size of each study (Table 1).

**TABLE 1:** Characteristics of all studies included in our analysis.

Authors (publication year)	Country	Data	Study period	Sample size of warfarin	Sample size of edoxaban		Mean ± SD age (years)		CHA_2_DS_2_-VASc score^a^		Follow-up duration
					Reduced dose	Regular dose	Warfarin	Edoxaban	Warfarin	Edoxaban	
Lee et al, (2018)	Korea	The National Health Insurance Service (NHIS) of Korea	January 2013 to December 2016	N = 12,183	N = 2,267	N = 1,794	70.7 **±** 10.5	70.3 ± 9.8	3.25 ± 1.72	3.22 ± 1.63	12 months
Chan et al, (2019)	Taiwan	The National Health Insurance Research Database (NHIRD)	June 2012 to December 2017	N = 19,761	N = 2,924	N = 1,653	74.6 ± 10.7	74.7 ± 1 0.8	3.6 ± 0.8	3.6 ± 1.6	16 months
Lee et al, (2019a)	Korea	The Korean National Health Insurance Service (NHIS)	January 2015 to December 2017	N = 25,420	N = 9,112	N = 6,384	71.2 ± 11.1	71.1 ± 10.4	3.60 ± 1.55	3.56 ± 1.41	12 months
Lee et al, (2019b) CrCl 80 > mL/min	Korea	The data from the Korean National Health Insurance Service (NHIS)	January 2014 to December 2016	N = 9,884	N = 560	N = 627	66.1 ± 11.5	65.8 ± 10.5	3.0 ± 1.9	3.0 ± 1.6	14 months
Kohsaka, et al, (2020)	Japan	Medical Data Vision database	March 2011 to July 2018	N = 19,905	N = 9,376	N = 3,216	76.1 ± 11.9	76.2 ± 10.8	3.8 ± 2.1	3.8 ± 2.0	24 months
Kwon et al, (2020) Age ≥ 80	Korea	The Korean Health Insurance Review and Assessment (HIRA) service	January 2015 to December 2017	N = 4,086	N = 2,928	N = 422	84.1 ± 3.6	84.1 ± 3.6	4.7 ± 1.1	4.7 ± 1.1	NA

^a^ CHA2DS2-VASc, indicates congestive heart failure, hypertension, age, diabetes mellitus, prior stroke, TIA, or thromboembolism, vascular disease, age, and sex.

The collected data also included the hazard ratios (HR) and 95% confidence intervals (CI) of edoxaban and warfarin, which were associated with each outcome of effectiveness (ischemic stroke) and safety (major bleeding). The Newcastle-Ottawa scale (NOS) was used to assess the quality of the studies (Wells et al., 2000) (Table 2). This scale comprised three sections of questions, and the highest possible sum was 9. Also, it is considering to be used for nonrandomized studies.

**TABLE 2:** Quality assessment of the included studies.

Study	Selection				Comparability		Outcome			Score*
	1	2	3	4	5a	5b	6	7	8	
Lee et al. (2018)	*	*	*	*	*	*	*	*	*	9
Chan et al. (2019)	*	*	*	*	*	*	*	*	*	9
Lee et al. (2019a)	*	*	*	*	*	*	*	*	*	9
Lee et al., 2019b CrCl 80 > mL/min	X	*	*	*	*	*	*	*	*	8
Kohsaka, et al. (2020)	*	*	*	*	*	*	*	*	*	9
Kwon et al., (2020) Age ≥ 80	X	*						X	X	6

*1. Representativeness of the exposed cohort; 2. Selection of the non-exposed cohort 3. Ascertainment of exposure; 4. Demonstration that the outcome of interest was not present at the start of the study; 5a. The study controls for Age, sex, and marital status; 5b. The study controlled for other factors; 6. Assessment of outcome; 7. Was the follow-up long enough for outcomes to occur; 8. Adequacy of cohort follow-up.

### 2.4 Statistical analysis

The HR and 95% CI were pooled from each study. The I^2^ test was used to evaluate the heterogeneity of the outcomes. A random-effects model was used to calculate the HR in our study. For each outcome, publication bias was measured statistically using the 2-tailed *p*-value results of Egger’s and Begg’s tests. A sensitivity analysis of excluding one trial at time has been conducted. Further sensitivity analysis was conducted based on the countries (Korea vs other countries) and the study quality (Low vs High). Data were analyzed using the Comprehensive Meta-Analysis Program (Biostat, Englewood, NJ, United States).

## 3 Results

**FIGURE 1:**
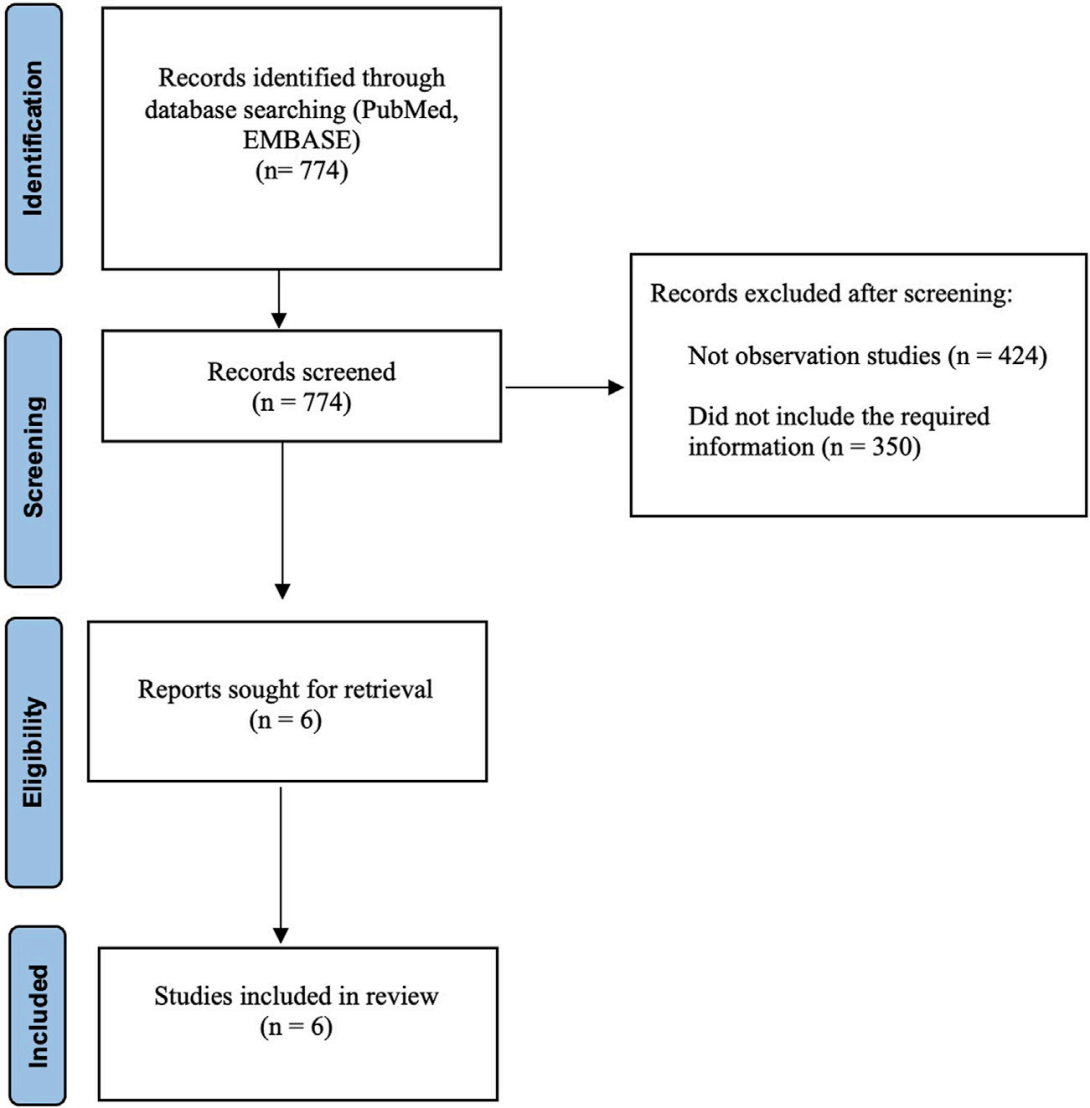
PRISMA flow diagram.

In our meta-analysis, we identified 774 studies which were retrieved from PubMed and EMBSE. We excluded 424 studies because they were not observational and 350 studies because they did not include the required information. Six retrospective observational studies were included in the final analysis (Figure 1). All included studies were of high quality.

### 3.1 Overview of all studies included in our analysis

Four studies were conducted in Korea, one in Japan, and one in Taiwan. In all studies, 87, 236 patients received warfarin, and 40,933 patients received edoxaban (Table 1). The effectiveness (ischemic stroke) and safety (major bleeding) outcomes were documented in all studies.

### 3.2 Outcomes of the study

#### 3.2.1 Ischemic stroke

**FIGURE 2:**
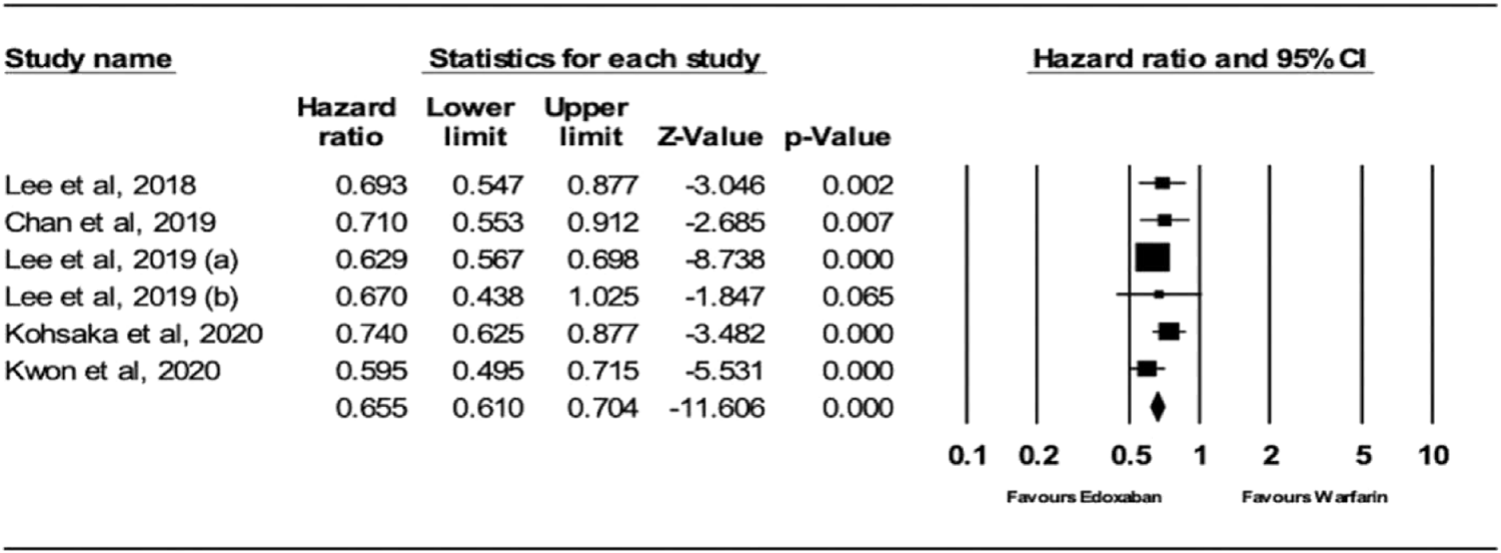
Forest Plot of the Effect of ischemic stroke on Edoxaban vs. Warfarin.

The analysis of the random effects model showed that patients receiving edoxaban had a significantly lower risk of ischemic stroke (IS) compared to warfarin (HR = 0.66; 95% CI, 0.61, 0.70; *p* < .0001), as shown in Figure 2. The I^2^ was 0% in the analysis, indicating that there was no heterogeneity between the studies.

#### 3.2.2 Major bleeding

**FIGURE 3:**
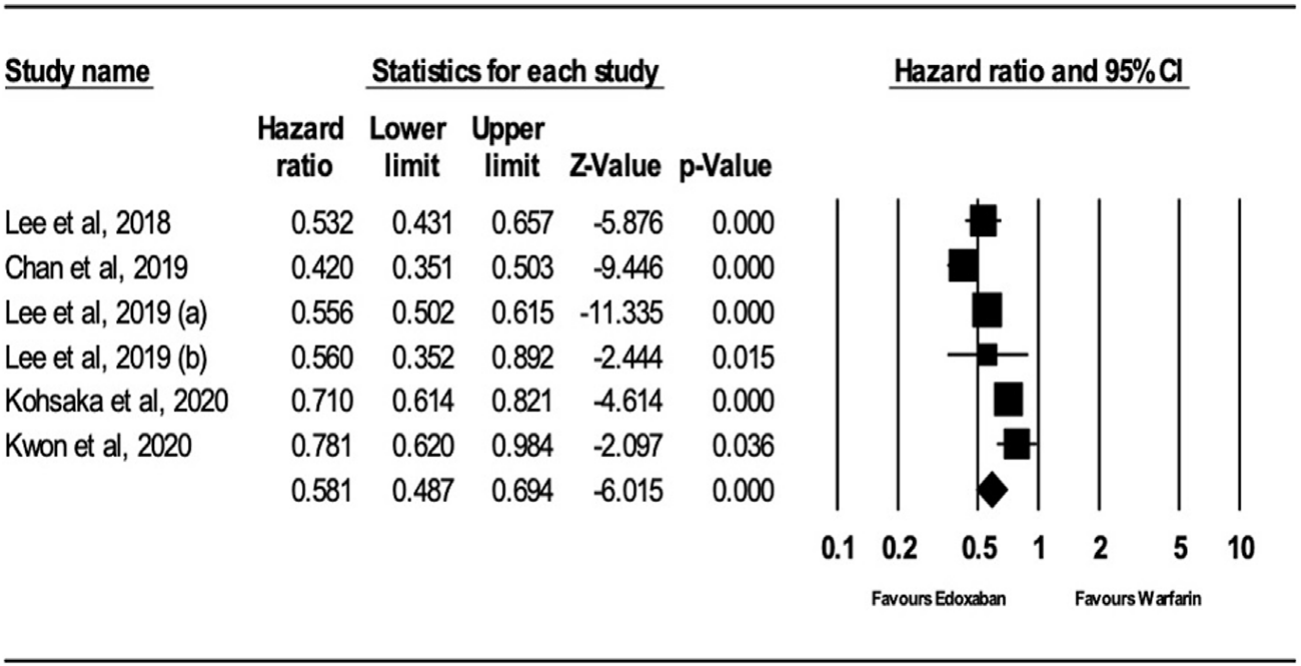
Forest Plot of the Effect of Major Bleeding on Edoxaban vs. Warfarin.

Analysis of the random-effects model demonstrated that patients receiving edoxaban had a significant reduction in major bleeding risk compared to those receiving warfarin (HR = 0.58; 95% CI, 0.49, 0.69; *p* < .0001), as presented in Figure 3. The I^2^ is 81.79% in the analysis, which means there is heterogeneity between the studies.

### 3.3 Sensitivity analysis

**FIGURE 4:**
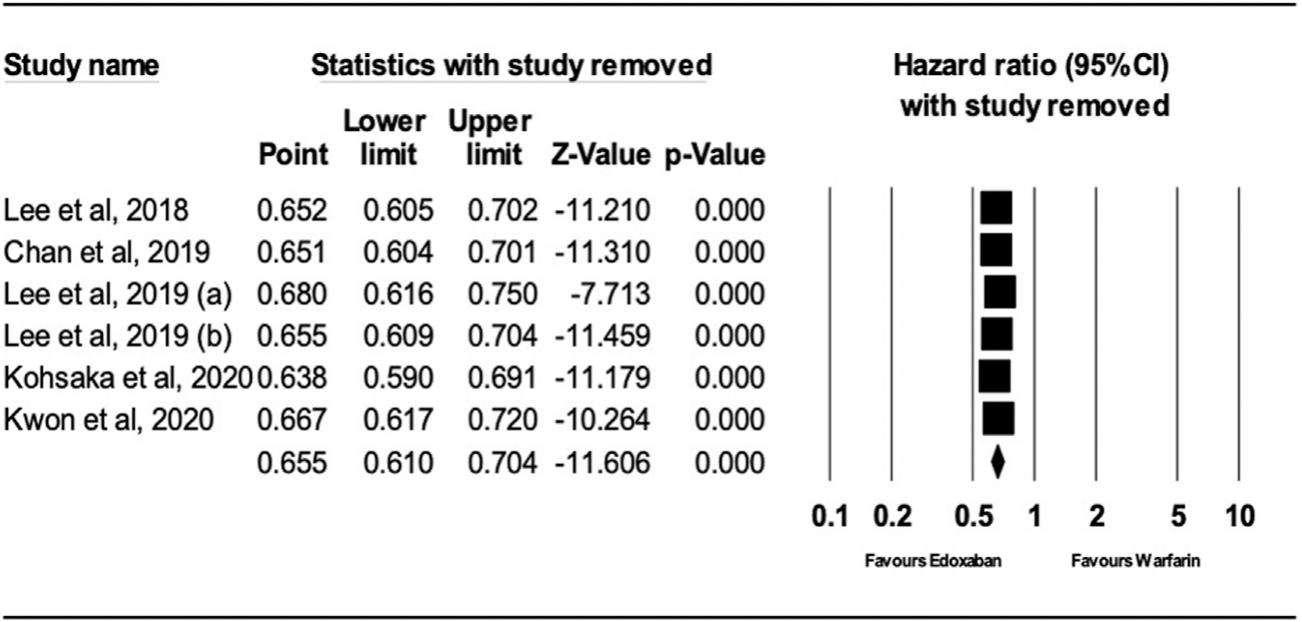
Forest Plot of the Effect of Ischemic Stroke on Edoxaban vs. Warfarin (sensitivity analysis).

**FIGURE 5:**
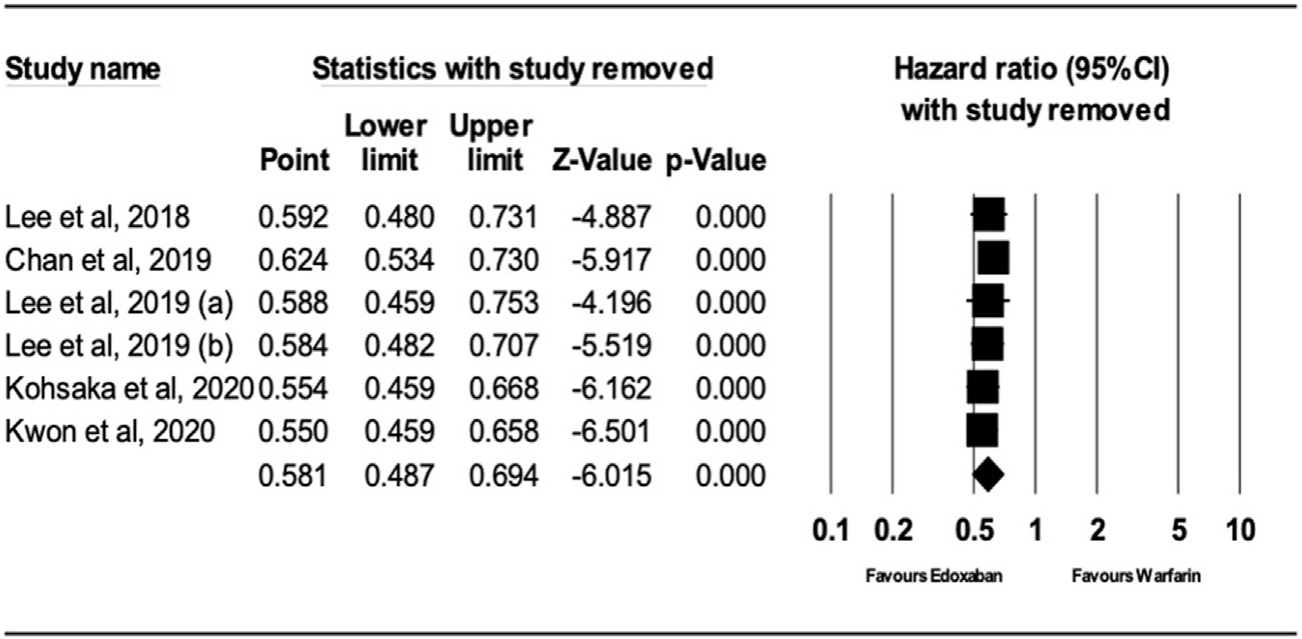
Forest Plot of the Effect of Major Bleeding on Edoxaban vs. Warfarin (sensitivity analysis).

After performing sensitivity analysis, the results for ischemic stroke and major bleeding using the random-effects model were HR = 0.66; 95% CI, 0.61, and 0.70 (P.0001) and HR = 0.58; 95% CI, 0.49, and 0.69 (P.0001), respectively, as shown in Figures 4, 5. In addition, the findings of the countries (Korea vs. other countries) and the study quality (Low vs. High) showed a better outcome of edoxaban compared to warfarin for both outcomes, as shown in Supplementary Figures S1–S8 (supplement section).

### 3.4 Publication bias assessment

The 2-tailed *p* values for Egger’s and Begg’s tests were >0.05, showing no significant evidence of publication bias for either outcome (ischemic stroke or major bleeding).

## 4 Discussion

In this updated comprehensive meta-analysis, we evaluated the risk of major bleeding and ischemic stroke in patients with NVAF treated with edoxaban *versus* warfarin by analyzing six real-world studies. We found that edoxaban performed better than warfarin in terms of major bleeding and ischemic stroke, as revealed by the sensitivity analysis.

According to a randomized controlled trial, in terms of avoiding strokes, edoxaban has a similar effect as warfarin and considerably decreases the risk of major bleeding and cardiovascular death (Giugliano et al., 2013). Moreover, an RCT found that patients with a history of major gastrointestinal bleeding (MGB) who regularly took edoxaban had a decreased risk of MGB compared to those who took warfarin; however, the risk of MGB was higher in patients taking a high dose of edoxaban than in those taking warfarin (Aisenberg et al., 2018). This is attributable to the inclusion criteria of the RCT or the racial makeup of the study population, as our study included only Asian patients. We were unable to differentiate between the different dose regimens because fewer patients used higher doses of edoxaban. Our findings revealed a lower risk of major bleeding in edoxaban users than in warfarin users.

Bai et al. compared the factor Xa inhibitors rivaroxaban and dabigatran with warfarin. In their meta-analysis, the risk of ischemic stroke was lower in the rivaroxaban group than in the warfarin group, which is consistent with the findings of our study, as rivaroxaban and edoxaban belong to the same drug category (Bai et al., 2017a). However, contrary to our findings, the risk of major bleeding was similar in the warfarin and rivaroxaban groups in their study. This could be due to the variation in time in the therapeutic range in various populations and real-world settings, which may result in poor warfarin (Wells et al., 2000; Singer et al., 2013; Piccini et al., 2014; Aisenberg et al., 2018; Lee et al., 2018; Lee et al., 2019a; Lee et al., 2019b; Chan et al., 2019; Kohsaka et al., 2020; Kwon et al., 2020).

A meta-analysis of RCTs comparing edoxaban with warfarin found a lower risk of major bleeding in edoxaban users than in warfarin users (Liang et al., 2021), which is consistent with our findings. Although their results are similar to our findings, our results are better because they are represented by HRs, which are better than the risk ratios (used in their study) in determining the event (George et al., 2020). Another meta-analysis of various CrCl levels found that edoxaban was associated with a lower risk of major bleeding than warfarin, which is consistent with our findings. They used a combination of different doses (30 and 60 mg). However, a major difference between our study and theirs is that they included both RCTs and retrospective studies, whereas our study focused only on retrospective studies. Moreover, their findings on ischemic stroke outcomes were different from ours, which may be related to their emphasis on various CrCl levels and different study designs (Wang et al., 2023). A network meta-analysis of major bleeding and ischemic stroke showed findings similar to those in this study. Although they used a network meta-analysis design, our meta-analysis included more studies than theirs (Kim et al., 2021).

Our study has several strengths. First, since our study was an inclusive, updated, and comprehensive meta-analysis, the total number of patients in all the included studies was 128,169, which is higher than that in other published meta-analyses (Kim et al., 2021; Liang et al., 2021; Wang et al., 2023), thus providing stronger evidence than other studies. Second, in all cohort studies included in our meta-analysis, the minimum follow-up periods were longer than those of RCTs (Liang et al., 2021), making our study more accurate in determining the outcome effect. Third, in contrast to RCTs that use predetermined standards, our analysis included observational studies that reflect real-world practice. Nonetheless, our study has several limitations. First, it represented only one geographic population in Asia; thus, our results cannot be generalized to other populations. Second, there was diversity in the age of our study samples; therefore, our results may not be representative of a specific age group, such as elderly patients. Third, we did not differentiate between various dosages of edoxaban, and most patients received low doses of edoxaban; therefore, our findings may be representative of low-dose edoxaban users. Fourth, most of the observation studies were based on the previously published AF guidelines. The updated AHA/ACC and ESC guidelines released new recommendation for CHA2DS2-VASc score and NVAF definition. Patients with NVAF and an elevated CHA2DS2-VASc score of 2 or greater in males or 3 or greater in female should be prescribed oral anticoagulants including DOACs to lower the risk of thromboembolic stroke. Regarding the new definition of NVAF, it is described as AF in a patient who does not have a mechanical heart valve or moderate to severe mitral stenosis (January et al., 2014; Heidbuche et al., 2015; J et al., 2019).

## 5 Conclusion

In summary, our updated comprehensive meta-analysis revealed that edoxaban effectively lowered the risk of major bleeding and ischemic stroke in patients with NVAF more effectively than warfarin. Therefore, healthcare professionals should weigh the benefits to their patients in terms of safety and efficacy when deciding between these two medications.

## Data Availability

The data analyzed in this study is subject to the following licenses/restrictions: We used Pubmed and EMBASE for our paper. Requests to access these datasets should be directed to MA, mmaalsultan@iau.edu.sa.
