# Supplementary figures and images for: Effectiveness and safety of edoxaban versus warfarin in patients with nonvalvular atrial fibrillation: a systematic review and meta-analysis of observational studies
Source: Front Pharmacol. 2023 Nov 16;14:1276491. doi: 10.3389/fphar.2023.1276491 (PMC10687440; doi:10.3389/fphar.2023.1276491)

**Supplementary section:**


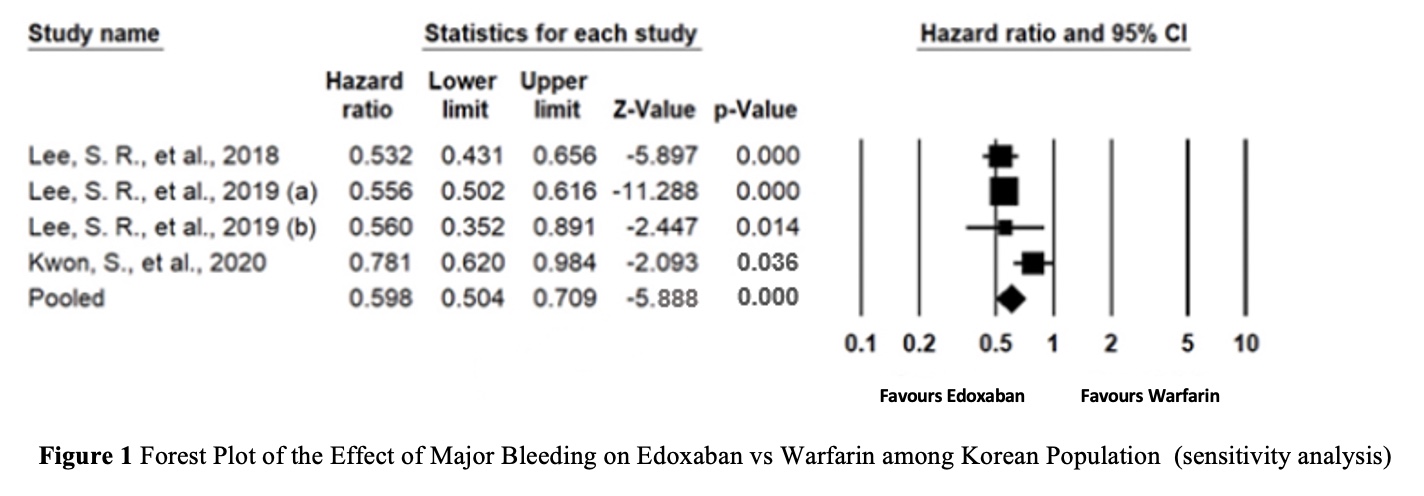


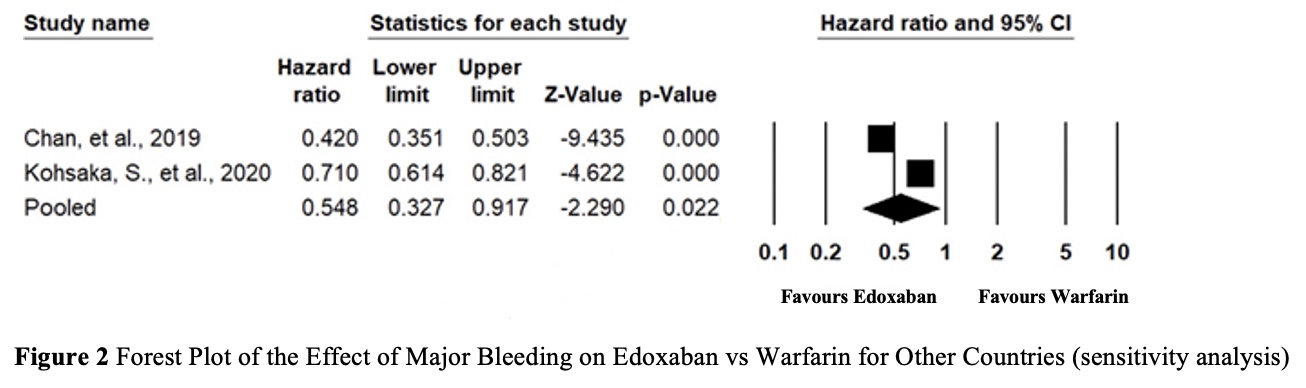


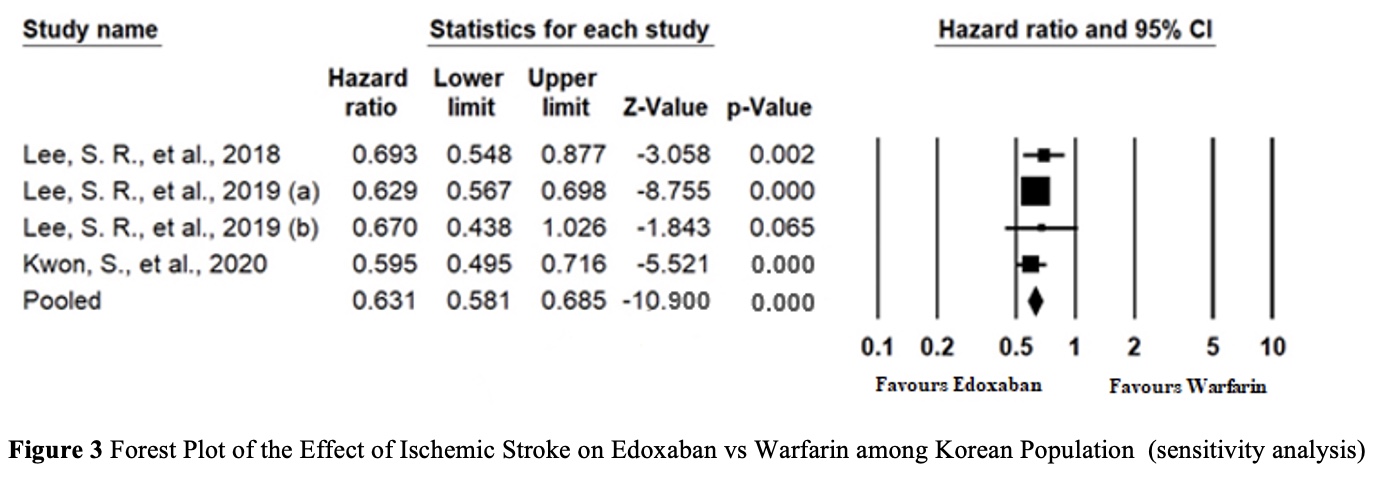


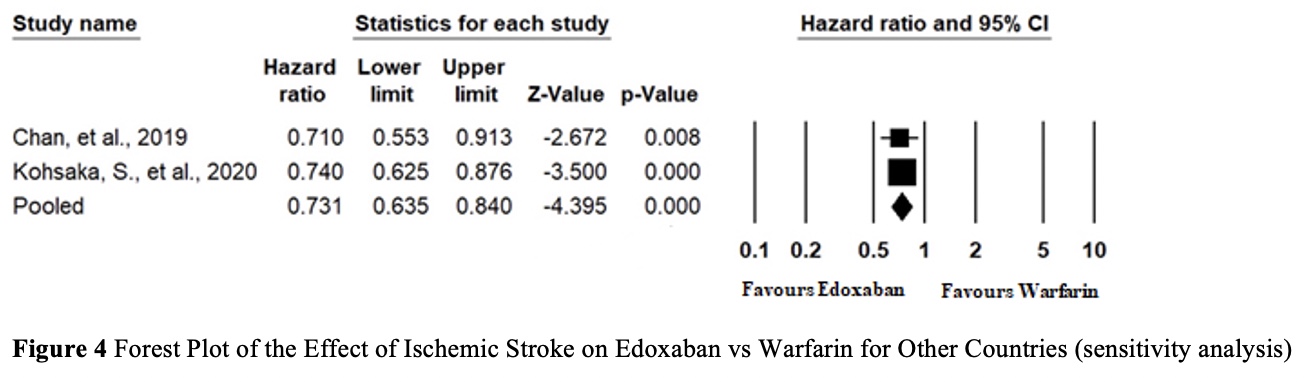


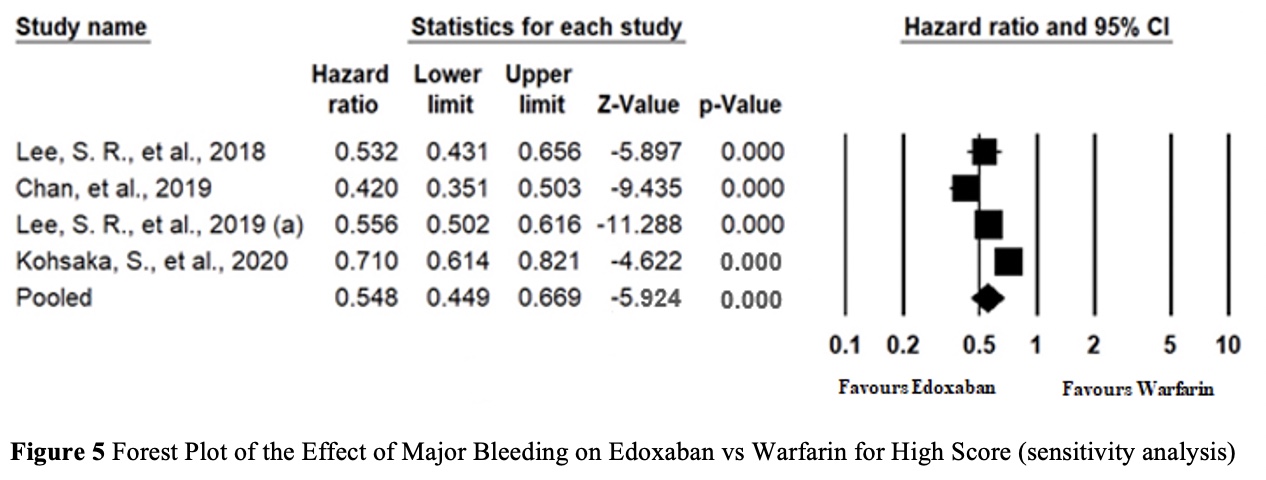

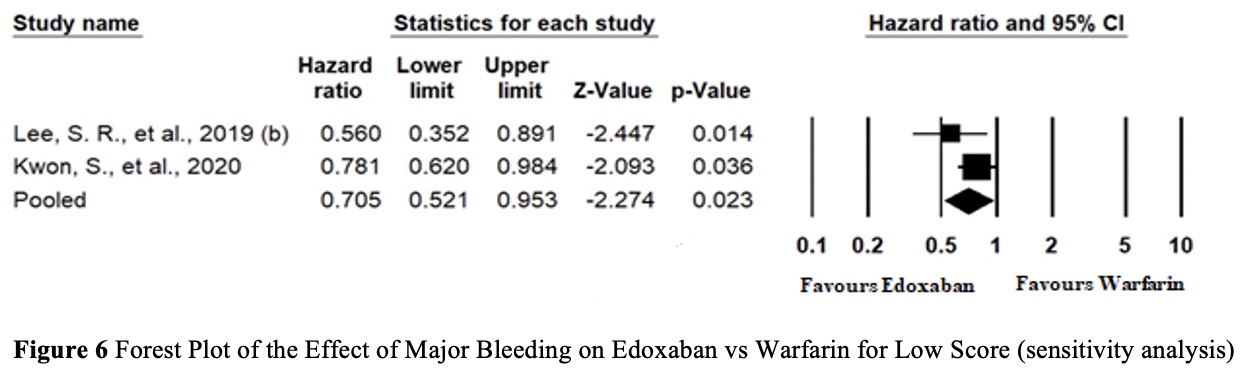


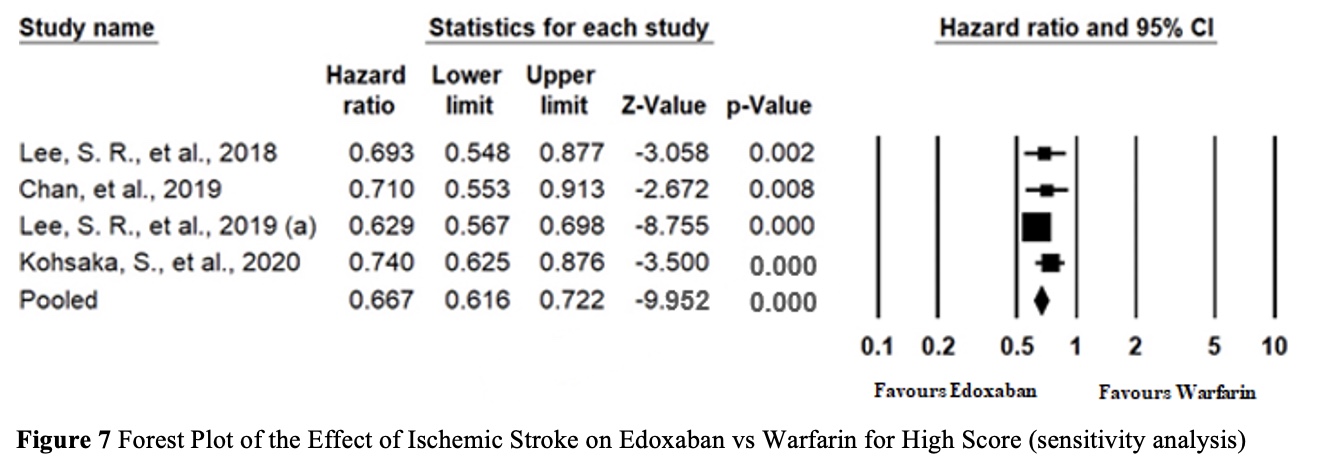


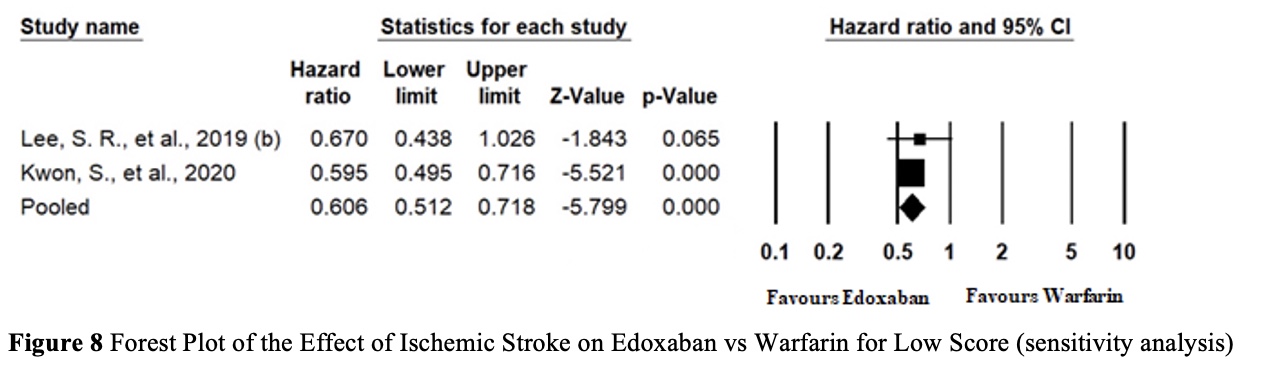

Supplement: Supplementary file 1 [file DataSheet1.docx]
